# Association rare d'un adenome pleomorphe et d'un carcinome epithelial myoepithelial de la glande parotide

**DOI:** 10.11604/pamj.2014.18.27.4203

**Published:** 2014-05-08

**Authors:** Ali Jahidi, Bouchaib Hemmaoui, Errami Noureddine, Darouassi Youssef, Rharrasi Issam, Chahdi Hafsa, Fouad Benariba

**Affiliations:** 1 Service ORL et CCF Hôpital Militaire d'Instruction Mohammed V, Rabat, Maroc; 2 Service d'Anatomo-Pathologie Hôpital Militaire d'Instruction Mohammed V, Rabat, Maroc

**Keywords:** Glande parotide, adénome pléomorphe, carcinome épiyhélial, myoépithélial, association, parotid gland, pleomorphic adenoma, epithelial carcinoma, myoepithelial, association

## Abstract

Le Carcinome épithélial - myoépithélial (CEM) est une tumeur maligne rare des glandes salivaires touchant principalement la glande parotide. Son association avec un adénome pléomorphe est exceptionnelle. Nous rapportons le cas d'une femme de 57 ans avec adénome pléomorphe de la glande parotide évoluant depuis plusieurs années. L'augmentation récente du volume de la glande associée à l'apparition d'adénopathies cervicales homolatérales nous a fait penser à une transformation maligne. L'examen histologique final après parotidectomie totale a montré une association inattendue d'un adénome pléomorphe et un CEM. Le CEM est une tumeur maligne de bas grade. Elle peut survenir de novo ou sur un adénome pléomorphe. La transformation maligne de l'adénome est suspectée devant l'augmentation rapide du volume de l'adénome avec apparition d'adénopathies cervicales. Toutefois, ces modifications cliniques peuvent annoncer l'apparition d'une tumeur distincte. Malgré sa tendance à la récidive locale et un faible potentiel métastatique, de rares cas de CEM peuvent avoir un comportement agressif et des métastases à distance. Le traitement consiste principalement en une résection chirurgicale complète si possible suivie d'une radiothérapie dans le but de prévenir la récidive locale.

## Introduction

Les tumeurs de la glande parotide sont rares. Elles représentent 1 à 4% des tumeurs de la tête et du cou. Les tumeurs bénignes sont les plus fréquentes dominées par l'adénome pléomorphe qui se aractérise par une évolution lente et un potentiel de dégénérescence maligne observé dans 3 à 14% des cas. Les Tumeurs malignes sont moins fréquentes, elles peuvent se développer de novo ou à partir d´un adénome pléomorphe. Parmi ces tumeurs, le carcinome épithélial myoépithélial (CEM) reste très rare et représente moins de 1% [1–3]. Longtemps considéré comme tumeur bénigne, le CEM est aujourd'hui reconnu comme entité distincte des tumeurs malignes de bas grade des glandes salivaires. Nous présentons un cas exceptionnel d'une patiente présentant un adénome pléomorphe évoluant depuis plusieurs années dont la modification récente des caractères cliniques et l'apparition d'adénopathies cervicales nous a d'abord fait penser à une transformation maligne avant de découvrir qu'il s'agit d'un carcinome épithélial myoépithélial apparu sur du tissu parotidien sain, signant ainsi une association rarement décrite à ce jour.

## Patient et observation

**Figure 1:**
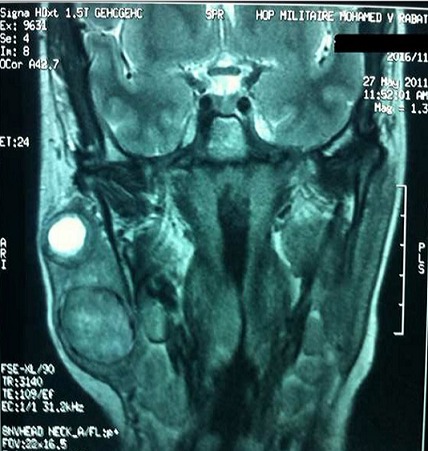
IRM en coupe coronale montrant les deux masses parotidiennes (vue 1)

**Figure 2:**
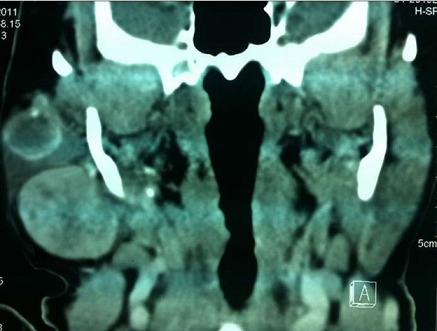
TDM en coupe coronale montrant les deux masses Parotidiennes (vue 2)

**Figure 3:**
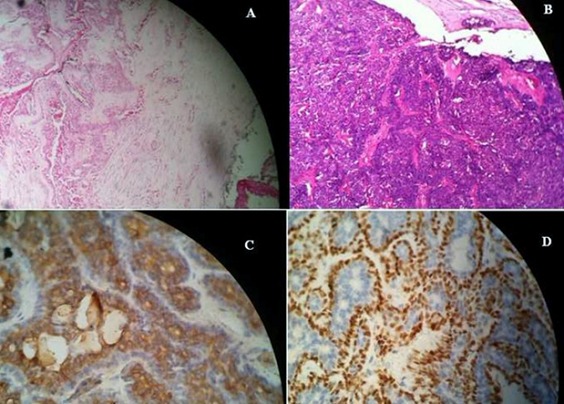
(A): coloration HE GX4 montrant l'adénome pléomorphe; (B): coloration HE Gx10 prolifération carcinomateuse à double composante épithéliale et myoépithéliale; (C): immunohistochimie expression des cellules épithéliales de l'anti CK AE1 AE3; (D): immunohistochimie expression des cellules myoépithéliales de l'anti P63

A.B est une patiente de 57 ans qui présente depuis 26 ans, une tuméfaction parotidienne. Les examens réalisés initialement notamment l’échographie et le scanner étaient en faveur d'un adénome pléomorphe. La patiente a refusé la chirurgie craignant les complications d'une parotidectomie. Il y a 2 ans la patiente a consulté pour une augmentation récente du volume de la tumeur, avec l'apparition d'une adénopathie cervicale homolatérale. Cette évolution nous a fait penser à une transformation maligne de l'adénome pléomorphe. L'IRM réalisée a mis en évidence la présence de deux masses parotidiennes la première mesurant 21x17 mm arrondie et bien limitée évoquant un adénome pléomorphe, la seconde, suspecte, mesurant 38x34x31mm polylobée avec rupture capsulaire(Figure 1). Le scanner trouve des adénopathies jugulo-carotidiennes supérieures droites dont la plus volumineuse mesure 29x17 mm (Figure 2) Une parotidectomie exofaciale a été réalisée, l'examen extemporané a révélé des signes de malignité sur le nodule suspect à l'imagerie. L'intervention a été poursuivie par une totalisation de la parotidectomie avec curage ganglionnaire cervicale homolatéral. Le résultat anatomopathologique définitif était surprenant : Il s'agissait d'une association de deux tumeurs parfaitement distinctes : un adénome pléomorphe (Figure 3 - A) et un carcinome épithélial myoépithélial caractérisé par la prolifération carcinomateuse à double composante (Figure 3 - B). Ce résultat a été confirmé par l´étude immunohistochimique qui met en évidence l´expression distincte des cellules épithéliales (Figure 3 - C) et myoépithéliales (Figure 3 - D). Les suites opératoires étaient simples. La patiente a bénéficié d'une radiothérapie à la dose de 50 grays sur la loge parotidienne et les aires ganglionnaires cervicales avec une bonne tolérance. Les contrôles réguliers n'ont retrouvé aucun signe de récidive locale ou loco-régionale.

## Discussion

Le CEM est une tumeur rare [1]. Il représente moins de 1% des tumeurs malignes des glandes salivaires. Il touche préférentiellement la glande parotide, mais des localisations au niveau des glandes sub mandibulaires, les glandes salivaires mineures ; voire extra orales ont aussi été décrites [4, 5] Le CEM a Longtemps été considéré comme une tumeur bénigne. La première description de cette tumeur a été faite par Donneth et col. [6]en 1972. Le CEM a été introduit dans la classification OMS des tumeurs salivaires en 1991 comme tumeur maligne de bas grade. La rareté de cette tumeur est aujourd'hui soulignée par plusieurs auteurs. [7, 8] Le CEM peut survenir de Novo ou sur un adénome pléomorphe [7]. La transformation est suspectée devant l'augmentation récente de la taille, l'apparition d'adénopathie, de douleurs ou de paralysie faciale. Toutefois cette évolution clinique peut signer l'apparition d'une tumeur maligne distincte de l'adénome sur du tissu parotidien sain. Notre observation illustre parfaitement ce cas rarissime. La présentation clinique du CEM n'est pas spécifique, il s'agit d'une masse indolore de croissance lente, la paralysie faciale est exceptionnelle. La femme est le plus souvent touchée entre 60 et 70 ans [7]. Macroscopiquement la tumeur est solide de couleur grise a la section, bien circonscrite, totalement ou partiellement encapsulée, [4] Microscopiquement, la tumeur est formée de deux populations cellulaires : au centre, une couche de cellules épithéliales à cytoplasme éosinophile et en périphérie une couche de cellules myoépithéliales claires. Cette double composante est confirmée par l’étude immuno histochimique [4]. L'IRM constitue l'examen de choix pour étudier les caractéristiques des tumeurs parotidiennes [5, 9]. La TDM avec injection de produit de contraste reste moins performante. L’échographie n'as que peu d'intérêt dans la pathologie tumorale parotidienne[5]. L'utilisation de l'IRM en diffusion pondérée est très prometteuse dans la différenciation tumorale [9]. La cytoponction à l'aiguille fine est un outil diagnostic important, mais le type de la tumeur n'est pas toujours déterminé avec précision[7]. Si la prise en charge de l'adénome pléomorphe parotidien est bien codifiée, il y a peu de données sur les différentes modalités de traitement du CEM ; l´exérèse chirurgicale large reste le traitement de choix [4]. La radiothérapie adjuvante peut-être utile pour prévenir la récidive locale. Le rôle de la chimiothérapie est incertain. [7]. L’évolution du CEM est marquée par la survenue de récidives locales dans 40% des cas apparaissant entre 9 mois et 20 ans. Les facteurs favorisants de la récidive sont: des marges positives, l'atteinte ganglionnaire, la nécrose tumorale et l'anaplasie myoépithéliale [7]. Des métastases à distance ont également été rapportées, les localisations les plus décrites sont les ganglions lymphatiques cervicaux, le poumon, le rein et le cerveau. Ces Métastases peuvent survenir très tard, même après dix ans de suivi [7]. Les taux de survie globale à Cinq ans et à 10 ans sont de 80% et 72%, respectivement [6]. Une longue période de surveillance est donc obligatoire, même pour des tumeurs opérées à un stade précoce avec résection chirurgicale complète.

## Conclusion

L'adénome pléomorphe de la parotide fait toujours craindre une transformation maligne d'autant plus que sa durée d’évolution est longue. Mais l'apparition d'une tumeur maligne indépendamment de l'adénome reste possible. Le CEM, tumeur rare, peut signer cette association exceptionnelle. Bien que le CEM soit considéré comme tumeur maligne de bas grade les différentes études ont montré la survenue de récidives locales parfois agressives et des métastases tardives à issue fatale. Il est donc recommandé d'avoir un suivi rigoureux pendant plusieurs années même si la tumeur est diagnostiquée à un stade précoce et sa résection est complète.
